# Exploring the Possibility of Medical Device Surveillance in Patients on Peritoneal Dialysis Using a Common Data Model

**DOI:** 10.3390/medicina61050814

**Published:** 2025-04-28

**Authors:** Seon Min Kim, Sooin Choi, You Kyoung Lee, Cheol Wan Lim, Byung Chul Yu, Moo Yong Park, Jin Kuk Kim, Seng Chan You, Seo Jeong Shin, Soo Jeong Choi

**Affiliations:** 1 Division of Nephrology, Department of Internal Medicine, Soonchunhyang University Bucheon Hospital, Soonchunhyang University College of Medicine, Bucheon 14584, Republic of Korea; 121746@schmc.ac.kr (S.M.K.); nephroybc@schmc.ac.kr (B.C.Y.); mypark@schmc.ac.kr (M.Y.P.); medkjk@schmc.ac.kr (J.K.K.); 2 Department of Laboratory Medicine and Genetics, Soonchunhyang University College of Medicine, Bucheon 14584, Republic of Korea; sooin2@schmc.ac.kr (S.C.); cecilia@schmc.ac.kr (Y.K.L.); 3 Department of General Surgery, Soonchunhyang University Bucheon Hospital, Soonchunhyang University College of Medicine, Bucheon 14584, Republic of Korea; cwlim@schmc.ac.kr; 4 Department of Biomedical Systems Informatics, Yonsei University College of Medicine, Seoul 03722, Republic of Korea; chandryou@yuhs.ac; 5 Institute for Innovation in Digital Healthcare, Yonsei University, Seoul 03722, Republic of Korea; lucid90sj@gmail.com

**Keywords:** peritoneal dialysis, equipment and supplies, databases, factual, electronic health records

## Abstract

*Background and Objectives*: Peritoneal dialysis (PD) requires well-functioning medical devices (MDs). PD complications can result in significant adverse events, including the discontinuation of PD, hospitalization, and death. This study aimed to evaluate the feasibility of detecting various PD complications and data related to MDs. *Materials and Methods*: A retrospective study was conducted on patients who received PD catheter insertions between January 2001 and March 2021 to evaluate PD-related complications. PD complications were evaluated through diagnostic, procedural, and MD codes using a common data model (CDM) and were compared with those from electronic health records (EHRs). The results from one CDM database were compared with those from another CDM database. *Results*: A total of 342 patients were enrolled. One hundred and ninety-five patients experienced PD complications more than once. Nineteen prescription codes and twenty diagnostic codes from the EHR were identified, covering 11 procedures, three MDs, and seven complications related to PD. Infectious complications were detected using the CDM, whereas mechanical complications were missed. Although data on PD catheters and adaptors were available in the EHR, they were not detected via the CDM. Some infectious and mechanical complications were identified via the CDM in the other database. After implementing amended matching, these data were detected. *Conclusions*: While some PD-related medical data recorded in EHRs were misrepresented or omitted during the CDM database extraction, transformation, and loading process, the CDM shows potential to serve as a source of real-world data for active surveillance.

## 1. Introduction

Peritoneal dialysis (PD) is a cost-effective therapy for treating kidney failure, offering advantages over in-center hemodialysis in terms of patient autonomy, reduced costs, and improved outcomes [1,2]. Well-functioning medical devices (MDs), such as PD catheters and transfer sets, are crucial for successful PD therapy. These devices enable adequate dialysis, and their proper function is associated with fewer complications, greater patient independence, and better clinical outcomes.

However, complications related to MDs can lead to significant adverse events, including the interruption or discontinuation of PD therapy, emergency department visits, hospitalizations, and the need for invasive procedures. Examples of such complications include catheter flow obstruction (also referred to as dysfunction or loss of patency), exit-site leaks, and abdominal pain, which are common PD catheter insertion-related issues.

Current surveillance of MD performance and complications relies largely on passive reporting, which is limited by its voluntary nature and the lack of comprehensive exposure data [3]. To address these limitations, there has been growing interest in using claims and electronic health record (EHR) data to evaluate MDs’ safety and effectiveness [4,5]. In 2015, the North American Chapter of the International Society for Peritoneal Dialysis established the North American PD Catheter Registry, a web-based platform to monitor patients undergoing laparoscopic PD catheter insertion [6]. This type of implant registry provides valuable information that enhances patient safety [7,8,9]. However, maintaining large registries can be challenging when complication rates are low and long-term follow-up is required.

An alternative approach for active surveillance is to use a common data model (CDM). A CDM is a logical and semantic data framework that standardizes multiple data sources into a common format, enabling more consistent data analysis [10]. To date, no studies have reported on CDM-based active surveillance of MD adverse events.

This study was designed as a preliminary investigation into the feasibility of using a CDM for active medical device surveillance. PD, which requires using an indwelling catheter as a core component, was selected as a representative model for evaluating MD-related complications.

## 2. Materials and Methods

The study protocol conformed to the ethical guidelines of the World Medical Association’s Declaration of Helsinki and was approved by the Institutional Review Board (IRB) of Soonchunhyang University Bucheon Hospital (SCHBC; IRB No. SCHBC 2021-05-002). The requirement for informed consent was waived by the IRB. This study adhered to the Strengthening the Reporting of Observational Studies in Epidemiology (STROBE) reporting guidelines [11].

### 2.1. Medical Records According to the Clinical Course of PD Patients

A retrospective chart review was conducted on patients who underwent PD catheter insertion between January 2001 and March 2021. The chart review included all patients aged 18 years or older who were undergoing follow-up in the nephrology division and underwent PD catheter insertion at SCHBC. Patients who had received PD catheter insertion at another hospital and transferred to our center were excluded. The review evaluated medical records, including PD-related procedures, MD usage, and complication diagnoses. Various procedures related to the overall course of PD were listed, and the corresponding electronic data interchange (EDI) codes were identified for each procedure. Among the PD-related MDs used at SCHBC, usage records for adapters, Tenckhoff catheters, and transfer sets were reviewed. The follow-up period was calculated from the insertion date of the initial catheter to the removal date or 30 June 2021, whichever came first. The occurrence of the following seven PD-related complications was reviewed: peritonitis, exit-site infection (ESI), tunnel infection (TI), outflow failure, peri-catheter leak, cuff shaving, abdominal herniation, and catheter cuff extrusion. Among these, peritonitis, ESI, and TI were classified as infectious complications, while outflow failure, peri-catheter leak, cuff shaving, abdominal herniation, and catheter cuff extrusion were classified as non-infectious (mechanical or procedure-related) complications.

### 2.2. CDM Mapping Status of Medical Records

**Figure 1:**
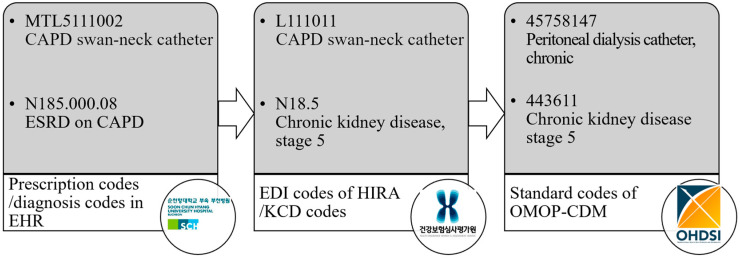
The process of transforming codes recorded in the hospital electronic health records to those of the common data model. CAPD, continuous ambulatory peritoneal dialysis; ESRD, end-stage renal disease; EHR, electronic health record; EDI, electronic data interchange; HIRA, Health Insurance Review and Assessment Service; KCD, Korean Standard Classification of Diseases; OMOP, Observational Medical Outcomes Partnership; CDM, common data model; OHDSI, Observational Health Data Sciences and Informatics.

After reviewing the medical records, diagnostic, procedure, and treatment material codes were evaluated for mapping to the CDM. SCHBC’s diagnostic coding is based on the Korean Standard Classification of Disease (KCD), which is adapted from the World Health Organization’s International Classification of Diseases (ICD) [12]. Lower-level codes are registered at a more detailed level than the minimum classification unit of the KCD due to requests from medical staff who prefer more precise diagnostic descriptions. The procedure and treatment material codes were institution-specific, corresponding to the EDI codes used by the Health Insurance Review and Assessment Service (HIRA). These data were converted to the source name “cdmpv531_0920_bucheon” using the Observational Health Data Sciences and Informatics open-source software and the Observational Medical Outcomes Partnership (OMOP) CDM version 5.3 database (Figure 1). At SCHBC, the CDM transformation was conducted as part of a government-supported national project through FeederNet, a centralized ETL service. The process was executed by an external partner company, and the complete mapping tables used during the ETL were not publicly disclosed.

The mapping status of CDM codes was assessed using OHDSI’s ATLAS tool. The appropriateness of ETL-converted codes was evaluated by examining their hierarchical structures and definitions through the Central Vocabulary Service, Athena (http://athena.ohdsi.org, accessed on 25 April 2025). For clinical events identified in the chart review but not mapped during the ETL process, appropriate SNOMED CT concepts were manually selected. Final mapping recommendations were made based on consensus among nephrologists and CDM experts at SCHBC.

### 2.3. Evaluation of the Adequacy of SCH Branches and Severance Databases for Monitoring PD Catheter-Related Adverse Events Leading to Peritonitis

Soonchunhyang (SCH) University operates four hospitals located in Seoul (SCHSU), SCHBC, Cheonan (SCHCA), and Gumi (SCHGM). These hospitals utilize a unified EHR system and share a standardized OMOP-CDM infrastructure, with CDM conversion conducted via the government-supported FeederNet ETL framework.

To evaluate whether the CDM databases of SCH branches can be effectively utilized to monitor the incidence of PD catheter-related adverse events, we conducted an analysis of PD catheter-related information and complication occurrences recorded in these databases. This involved performing a comparative analysis, in which data from SCHBC were compared with those of the other branches—SCHSU, SCHCA, and SCHGM—to assess PD catheter utilization and recorded instances of complications among patients with PD procedure records.

For external validation to explore the generalizability of the CDM surveillance approach, an identical query was applied to the CDM database of Severance Hospital. Severance Hospital employs a distinct EHR system and performs CDM conversion through an internally developed ETL pipeline independent of FeederNet.

## 3. Results

### 3.1. Clinical Course of PD

**Figure 2:**
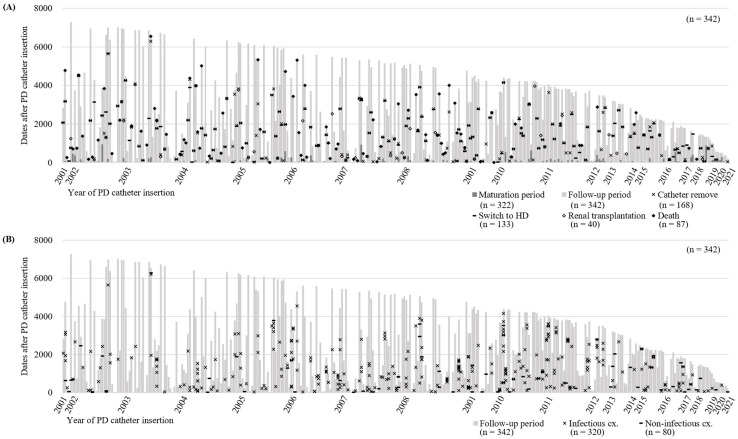
Clinical course (**A**) and complication occurrence (**B**) of 342 patients with PD catheterization from 2001 to 2021. PD, peritoneal dialysis; HD, hemodialysis; cx., complication.

A total of 342 patients who underwent PD catheter insertion at SCHBC were reviewed. Among them, 21 patients did not initiate PD, while 321 patients proceeded with the externalization of the PD catheter and began PD after a mean period of 74.6 days. During the follow-up period of 1378.0 ± 95.1 days, 168 patients had their catheters removed (Figure 2A). A total of 133 patients transitioned to hemodialysis, and 40 patients underwent kidney transplantation. Among these patients, 87 died (Figure 2A). Of the 342 patients, 195 experienced complications more than once, with 320 cases of infectious complications and 80 cases of non-infectious complications reported (Figure 2B). Infectious complications, including 257 cases of peritonitis, 55 ESIs, and eight tunnel infections, were recorded in 162 patients. Eighty non-infectious complications occurred in 65 patients. Cuff shaving, herniorrhaphy, and leak management were the most frequently performed interventions.

### 3.2. PD-Related Codes Recorded in EHR

**Figure 3:**
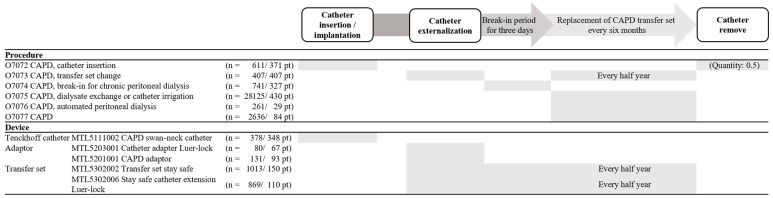
Current status of EHR data for peritoneal dialysis catheter. CAPD, continuous ambulatory peritoneal dialysis; EHR, electronic health record; pt, patients.

Among the 11 procedures related to the overall course of PD, only six EDI codes were extracted (Figure 3 and Supplementary Table S1, only online). Additionally, only five codes were identified among the 12 nationwide therapeutic materials for PD-related MDs. Eight diagnostic codes related to PD complications were recorded at SCHBC.

Figure 3 illustrates the clinical progress of PD and the records of PD-related procedures and MD EDI codes. Although catheter insertion and removal are distinct medical procedures, the O7072 code was used for both because no separate EDI code exists for catheter removal. Consequently, catheter removal was recorded under the O7072 code with a claim quantity of 0.5 to account for the difference in procedure complexity. While transfer set changes were performed every 6 months or when complications occurred, the same code (O7073) was used for both routine and complication-driven changes. The code for transfer sets (MTL5302002 or MTL5302006) was entered along with the procedure code (O7073) every 6 months, while the code for the adapter (MTL5203001 or MTL5201001) was only recorded when initially replacing of the CAPD transfer set. The code O7075, which is no longer used, was the most frequently entered code until June 2017, with 28,125 entries in 430 patients. Codes O7076 and O7077 have been in use since July 2017.

### 3.3. Comparison of First Complication Events in SCHBC via Chart Review and the CDM (Table 1)

Prior to CDM-based analysis, the seven PD-related complications identified through chart review were examined for mapping status and potential omissions within the existing ETL structure. Recommended mappings were proposed through consensus between nephrologists and CDM experts at SCHBC (Supplementary Table S2, only online).

**Table 1:** Comparison of initial complication count by chart review and common data model.

Complications	Diagnosis	Chart Review (*n* = 321)	CDM (*n* = 314)		Detection Proportion ^‡^ (%)
Infectious complications	Peritonitis	132 (41.1)	127 (40.4)	173 * (55.1)	96.2
	Exit-site infection	41 (12.8)	0 (0.0)		0
	Tunnel infection	8 (2.5)	0 (0.0)		0
Non-infectious complications	Granulation	9 (2.8)	0 (0.0)		0
	Cuff shaving	14 (4.4)	0 (0.0)		0
	Leak	12 (3.7)	0 (0.0)	33 ^†^ (10.5)	0
	Reposition	9 (2.8)	0 (0.0)		
	Malfunction	11 (3.4)	0 (0.0)		
	Herniorrhaphy	13 (4.0)	13 (4.1)		100
	Appendectomy	1 (0.3)	0 (0.0)		0
	Pleural–peritoneal shunt	1 (0.3)	0 (0.0)		0
	Abdominal pain	3 (0.9)	0 (0.0)		0
	Tear	3 (0.9)	0 (0.0)		0
	Pancreatitis	4 (1.2)	0 (0.0)		0
Death		82 (25.5)	89 (28.3)		108.5

CDM, common data model. Data are presented as *n* (%). * Number of patients with 440,276 (infection and/or inflammatory reaction due to internal prosthetic device, implant and/or graft) codes; ^†^ Number of patients with 43,021,974 (complication associated with device) codes.; ^‡^ Detection proportion (%) was calculated as proportion of CDM-detected cases over chart review cases for each complication.

A comparative analysis between chart review and CDM-based detection revealed substantial discrepancies, particularly in identifying non-infectious complications. While 127 of 132 peritonitis cases (96.2%) were detected using the CDM, none of the 41 exit-site infections or eight tunnel infections identified via chart review were captured. Similarly, for non-infectious events, such as cuff shaving, leakage, and catheter malfunction, all cases identified through manual chart review were undetected via the CDM. Among the complications evaluated, only herniorrhaphy showed full concordance, with 13 cases reported in both datasets, yielding a detection proportion of 100%. The CDM identified more deaths than the chart review.

### 3.4. Comparative Analysis of PD Catheter-Related Complications Across SCH Branch Hospitals and Severance Hospital

To assess the consistency of CDM-based surveillance across institutions, we conducted a cross-hospital comparison using the same query applied in SCHBC. The query was carried out in three other Soonchunhyang University branches (SCHSU, SCHCA, SCHGM), all of which share the same EHR system and underwent CDM conversion via FeederNet.

The number of patients with PD procedure records was highest at SCHBC, with 427 individuals, followed by SCHSU and SCHCA, with 350 and 300 individuals, respectively. SCHGM had the fewest, with 53 individuals. SCHBC also recorded the highest number of PD catheter insertion cases, with 366 instances, representing 85.7% of patients with PD procedure records. SCHBC also documented 315 cases of swan-neck catheter prescriptions, accounting for 73.8% of its PD procedure population. In contrast, SCHSU recorded 117 cases, representing 33.4% of its PD procedure group. SCHCA and SCHGM reported no cases of swan-neck catheter prescriptions. Therefore, complication occurrence data were based on PD procedures rather than catheter prescriptions in SCHCA and SCHGM.

The incidences of peritonitis, mechanical complications, and hernias following PD procedures were documented and compared. SCHBC had the highest number of peritonitis cases, with 130, representing a 30.4% incidence. SCHSU and SCHCA reported 102 and 84 cases, with incidences of 29.1% and 28.0%, respectively. SCHGM had the lowest with 12 cases, representing a 22.6% incidence among its PD procedure population.

For external validation, the query was also carried out at Severance Hospital, which uses a different EHR and an internally developed CDM transformation pipeline. Unlike at SCH University Hospital branches, specific PD catheter insertion codes were not identified. However, codes for associated complications, both infectious and non-infectious, were available (Table 2).

The incidences were calculated using the number of patients with PD procedure records at each institution. SCHBC was used as the reference group for statistical comparisons. Several statistically significant differences were observed between SCHBC and the other hospitals. Within the SCH network itself, significant differences in the incidences of peritonitis and mechanical complications were observed (*p* < 0.05), despite all institutions sharing the same EHR system and undergoing CDM conversion through the FeederNet-based ETL process. The incidences at Severance Hospital exhibited significant differences in all complications.

## 4. Discussion

Real-world data, such as electronic medical records (EMRs) and claims data, are widely used in observational studies. However, many EMR datasets are inconsistent across hospitals and can be difficult to access, making large-scale research challenging. One proposed solution is the OMOP-CDM, which has been adopted by many hospitals for standardization [13,14,15].

Research using the CDM can allow access to large datasets with minimal effort and cost. Additionally, CDM provides anonymized data by removing personally identifiable information, ensuring privacy protection.

Some researchers have demonstrated that analyzing EMR data using standardized methods is cost-effective and supports proactive surveillance by physicians. This study builds upon previous research, showing that even when standardized through the CDM, EMR data can be utilized for the active surveillance of MD adverse events [16]. While this study demonstrated the feasibility of using the CDM for the active surveillance of specific limited MDs, such as PD catheters, it also revealed important limitations, despite some encouraging results in detecting clearly defined or well-documented clinical events.

In this study, a comparison between chart review and the CDM data at our institution revealed that peritonitis was captured at a notably higher proportion than other complications. In addition, procedures with clear billing codes, such as herniorrhaphy and well-defined clinical events including death, were captured in the CDM with a level of completeness similar to, or in some cases exceeding, that of the chart review. These results suggest that even when ETL limitations are present, the CDM can reliably reflect clinical reality for events that are clearly defined and closely tied to reimbursement or regulatory requirements. In such cases, the EHR records tend to be more complete and standardized, allowing for the more accurate representation of such events in the CDM following the ETL process.

The limitations identified in our study point to several structural challenges that must be addressed for the CDM to become a more reliable tool. We propose the following considerations for improving the effectiveness of CDM-based surveillance systems.

### 4.1. Limitations of Diagnosis-Based Analysis

One primary limitation of CDM-based analysis is its reliance on explicitly entered structured data. However, diagnostic codes, such as ICD or country-specific modifications including KCD, cannot capture all clinical scenarios. For example, “tunnel infection” does not exist under the general ICD/KCD categories. To represent such conditions, SCH branches use more detailed, subdivided codes (e.g., T857.004.01). In this case, two additional levels are appended to the standard T85.7 code: the first for the device type (004) and the second for the specific clinical context (01). These institution-specific codes are not standardized across hospitals and are often lost during the ETL process if only the primary category (e.g., T857) is mapped.

Furthermore, even when diagnostic codes are highly detailed, variability in code selection by clinicians remains a significant issue. The more detailed a coding system is, the more likely clinicians are to omit entering specific diagnostic codes altogether due to complexity or inconvenience. Additionally, when diagnostic code entry is not mandated by reimbursement requirements or quality assessments, clinicians may prefer to document patient conditions only in unstructured text or image-based records rather than entering structured codes.

### 4.2. Limitations of Procedure and Prescription Coding (EDI Codes in Korea)

Another key limitation lies in the use of procedure and prescription codes based on electronic data interchange (EDI). Ideally, every medical prescription and procedure would have a uniquely identifiable code, allowing for accurate mapping during the ETL process. However, the Korean reimbursement code system does not cover all procedures. For instance, while there is an EDI code for PD catheter insertion, no corresponding code is available for its removal (Figure 3 and Supplementary Table S1, only online). At our institution, we have developed a makeshift solution by recording insertion as 1 and removal as 0.5 in the EHR. Similarly, procedures related to PD catheter irrigation and cuff shaving lack EDI codes. While these procedures may be documented in unstructured formats, such as operative notes or progress reports, they are typically not captured during CDM transformation due to their text-based or image-based format. Given the CDM’s reliance on structured data, such omissions introduce significant blind spots in device-related surveillance and procedural analytics.

### 4.3. Limitations of ETL Accuracy

Although tools such as OHDSI/Achilles provide high-level validation of CDM transformations, they are insufficient for evaluating the appropriateness of individual clinical mappings. As discussed in the previous sections on diagnosis and procedure codes, the accuracy of ETL processes largely depends on how precisely structured codes and test results are entered into the EHR. In many cases, institutions use non-standardized, highly localized codes that are ultimately truncated or aggregated into broader categories during ETL. As a result, even if these codes exist in standardized vocabularies, they may not be accurately retained when transformed from ICD/KCD or local coding systems.

Ensuring accurate ETL mappings often requires active participation and oversight by clinical experts familiar with the source data and medical context. However, most researchers and clinicians are not directly involved in the ETL processes. In Korea, where CDM transformation is frequently outsourced to government-supported contractors, the mapping logic and vocabulary crosswalks are rarely disclosed. This restricts the ability to audit or revise mappings, as any changes typically require formal project-based updates through external vendors.

While reducing excessive code granularity could mitigate some issues, clinical accuracy may be compromised. Therefore, when detailed local codes are used, clinicians actively engaged in real-world care and documentation should participate in mapping to ensure the clinical validity of the transformation.

### 4.4. Limitations of EDI Coding and the Importance of UDI Integration

Another important limitation of the current CDM implementation is the lack of detailed device-specific information within EDI-based coding structures [17,18]. Although the present study broadly categorized devices under terms such as “PD catheter”, it was not possible to determine which specific product was used or to trace its corresponding lot number. This level of granularity is critical for effective post-market surveillance of medical devices.

The unique device identifier (UDI) system provides a structured solution to this issue. The UDI-DI (device identifier) refers to the specific product and manufacturer, while the UDI-PI (production identifier) includes traceability information such as the lot number or expiration date [19]. In Korea, the documentation of UDIs is mandated for implantable devices (Supplementary Figure S1, only online). However, these data have not yet been routinely transformed into the CDM. If UDI fields were consistently integrated into CDM datasets, it would significantly enhance device-level and lot-level tracking and enable more robust active surveillance strategies. Such integration would allow researchers to evaluate complication rates by device model or manufacturing batch, enabling the earlier detection of device-related safety signals [20].

### 4.5. Limitations in Cross-Institutional Comparisons

This study also highlights the limitations of cross-institutional comparisons within CDM-based surveillance. Differences in diagnostic coding practices, the use of non-standardized institution-specific codes, and variability in ETL execution across hospitals significantly hinder interoperability. It becomes difficult to determine whether observed differences in complication rates reflect true clinical variation, differences in patient populations, or inconsistencies introduced during the ETL process.

Although tools such as Achilles and DQD are available to evaluate the overall ETL quality, they are insufficient for detecting granular inconsistencies in code-level transformations [21,22]. Without access to detailed mapping logic and EHR-level data before anonymization, validating institutional ETL accuracy remains a challenge. To enhance reliability, future multi-institutional analyses should consider adopting intermediate tools or audit systems that allow for partial matching between original EHR data and transformed CDM data prior to full anonymization.

Until such validation tools become widely available, we suggest that active surveillance efforts focus primarily on within-institution monitoring. In these settings, device-specific trends, such as categorizing complication rates by model or lot number, can be more reliably assessed without the confounding factors introduced by inter-hospital heterogeneity.

Given the current limitations in EHR documentation, data standardization, and CDM transformation, the use of the CDM for comprehensive medical device surveillance remains constrained. However, for well-documented events such as death, CDM-based analyses can still offer meaningful insights. With future improvements, such as standardized UDI integration, enhanced diagnosis coding practices, the expansion of procedure codes, and stronger policy-driven incentives for accurate documentation, the utility of the CDM as a real-world data infrastructure could be substantially improved across a broader range of clinical scenarios.

## 5. Conclusions

In conclusion, while the present environment imposes several limitations on using the CDM for active medical device surveillance, its potential should not be overlooked. With the continued development of EHR documentation standards, ETL transparency, and the nationwide adoption of structured UDI data, the CDM can evolve into a more robust and reliable platform for post-market device monitoring and real-world evidence generation.

## Figures and Tables

**Table 2:** Evaluation of peritoneal dialysis catheter-related complication incidences across the Soonchunhyang Branch and Severance Hospitals.

	SCHBC	SCHSU	SCHCA	SCHGM	Severance
Data loading year	2001–	2003–	2006–	2007–	2006–
Number of persons in records	1,301,116	1,098,041	902,797	586,891	5,773,561
Patients with PD procedure records	427	350	300	53	1796
Patients with PD catheter insertion records	366	208	216	42	
Patients prescribed swan-neck catheter	315	117	0	0	596
PD patients experiencing peritonitis	130	102	84 *	12 *	253
PD patients experiencing mechanical complications	73	59	116 *	8 *	102
PD patients experiencing a hernia operation	18	5	9 *	1 *	36

PD, peritoneal dialysis; SCHBC, Soonchunhyang University Bucheon Hospital; SCHSU, Soonchunhyang University Seoul Hospital; SCHCA, Soonchunhyang University Cheonan Hospital; SCHGM, Soonchunhyang University Gumi Hospital. * Records were not taken with a PD catheter device but with PD procedure codes.

## Data Availability

The data that support the findings of this study are available on request from the corresponding author.

## Supplementary Materials

The following supporting information can be downloaded at: https://www.mdpi.com/article/10.3390/medicina61050814/s1, Table S1: Current Status and Improvement of CDM Standard Terminology Mapping of PD Related Codes; Table S2: Comparison of Term/Codes between SNOMED, Study Institute, and Mapped OMOP-CDM; Figure S1: Electronic health record data of peritoneal dialysis catheter using unique device identification scanning.

## Abbreviations

The following abbreviations are used in this manuscript:

PD	Peritoneal dialysis
MD	Medical device
CDM	Common data model
EHR	Electronic health records
IRB	Institutional Review Board
STROBE	Strengthening the Reporting of Observational Studies in Epidemiology
EDI	Electronic data interchange
HIRA	Health Insurance Review and Assessment Service
KCD	Korean Standard Classification of Disease
CAPD	Continuous ambulatory peritoneal dialysis
ESRD	End-stage renal disease
OMOP	Observational Medical Outcomes Partnership
OHDSI	Observational Health Data Sciences and Informatics
SCH	Soonchunhyang
SCHBC	Soonchunhyang University Bucheon Hospital
SCHSU	Soonchunhyang University Seoul
SCHCA	Soonchunhyang University Cheonan
SCHGM	Soonchunhyang University Gumi
HD	Hemodialysis
EMR	Electronic medical records
ICD	International Classification of Diseases
ESI	Exit-site infection
TI	Tunnel infection
ETL	Extract, transform, and load
UDI	Unique device identification
UDI-DI	UDI-device identifier
UDI-PI	UDI-production identifier
