# Supplementary material for: Exploring the Possibility of Medical Device Surveillance in Patients on Peritoneal Dialysis Using a Common Data Model
Source: Medicina (Kaunas). 2025 Apr 28;61(5):814. doi: 10.3390/medicina61050814 (PMC12113496; doi:10.3390/medicina61050814)
Supplement: Supplementary file 1 [file medicina-61-00814-s001.zip › medicina-3579056-supplementary.pdf]

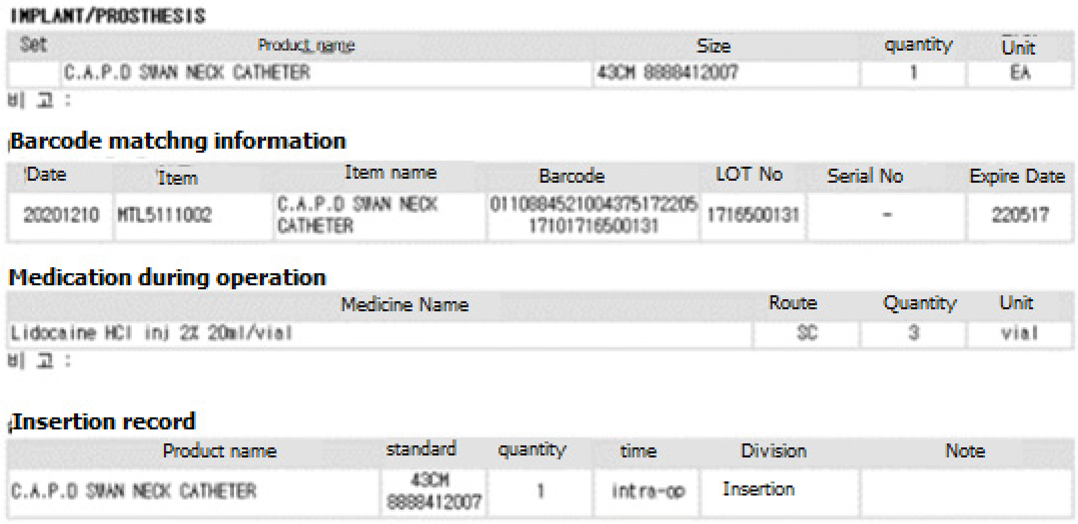

IMPLANT/PROSTHESIS

Set	Product name	Size	quantity	Unit
	C.A.P.D SWAN NECK CATHETER	43CM 8888412007	1	EA

비 고 :

Barcode matchng information

Date	Item	Item name	Barcode	LOT No	Serial No	Expire Date
20201210	MTL5111002	C.A.P.D SWAN NECK CATHETER	011088452100437517220517101716500131	1716500131	-	220517

Medication during operation

Medicine Name	Route	Quantity	Unit
Lidocaine HCl inj 2% 20ml/vial	SC	3	vial

비 고 :

Insertion record

Product name	standard	quantity	time	Division	Note
C.A.P.D SWAN NECK CATHETER	43CM 8888412007	1	intra-op	Insertion	

Supplementary Figure S1. Electronic health record data of peritoneal dialysis catheter using unique device identification scanning.

Supplementary Table S1. Current status and improvement of CDM standard terminology mapping of PD related codes

Classification	Electronic health record	Current CDM (by FEEDER-NET)			Improved code mapping		
	Code name	ID name	Concept	Vocabulary	ID name	Concept	Vocabulary
PD related procedures							
Insertion of CAPD catheter	O7072 CAPD, catheter insertion	4108838 Insertion of chronic ambulatory PD catheter	Standard	SNOMED	Maintain		
Implantation of CAPD catheter					No suitable code		
Replacement of CAPD transfer set	O7073 CAPD, transfer set change	4235076 Replacement of PD catheter	Standard	SNOMED	37016665 Replacement of CAPD transfer set	Standard	SNOMED
Break-in	O7074 CAPD, break-in for chronic PD	4156957 Chronic ambulatory PD catheter procedure	Standard	SNOMED	4030832 Flushing of chronic ambulatory PD catheter	Standard	SNOMED

Dialysate exchange						46271816 Management of PD	Standard	SNOMED
Flushing (catheter irrigation)		O7075 CAPD, dialysate exchange or catheter irrigation	46271816 Management of PD	Standard	SNOMED	4030832 Flushing of chronic ambulatory PD catheter	Standard	SNOMED
Catheter maintenance						4247794 PD catheter maintenance	Standard	SNOMED
Automated PD		O7076 CAPD, automated PD				4323627 Automated PD	Standard	SNOMED
CAPD		O7077 CAPD	4080169 CAPD	Standard	SNOMED	Maintain		
Cuff shaving		NA	NA			4231646 Shaving of PD catheter cuff	Standard	SNOMED
Removal of CAPD catheter		O7072 CAPD, catheter insertion (amount: 0.5)	4108838 Insertion of chronic ambulatory PD catheter	Standard	SNOMED	4031777 Removal of PD catheter	Standard	SNOMED
PD related device								
Adaptor	Silicone	MTL5203001 Catheter adapter Luer-lock	42089706 Catheter adapter Luer-lock	Non-standard	EDI	45771583 PD catheter adaptor	Standard	SNOMED
		NA Stay safe catheter adaptor	42100904 Stay safe catheter adaptor	Non-standard	EDI			
	Titanium	MTL5201001 CAPD adaptor	42089102 CAPD adaptor	Non-standard	EDI			
		NA CAPD catheter adapter (TA200)	42089576 CAPD catheter adapter (TA200)	Non-standard	EDI			
Tenckhoff catheter	Straight	NA CAPD peritoneal tenckhoff catheter	42089104 CAPD peritoneal tenckhoff catheter	Non-standard	EDI	45757931 PD catheter, acute/chronic	Standard	SNOMED
	Swan-neck curl (mixed type)	NA PD catheter curl cath	42098087 PD catheter curl cath	Non-standard	EDI			
	Swan-neck or curl	MTL5111002 CAPD swan-neck catheter	42089105 CAPD swan-neck catheter	Non-standard	EDI	45758147 PD catheter, chronic	Standard	SNOMED
		NA CAPD swan-neck coil-catheter	42089106 CAPD swan-neck coil-catheter	Non-standard	EDI			
Transfer set	24 hr	NA PD-PAED plus	42098235 PD-PAED plus	Non-standard	EDI	45758301 PD system tubing set	Standard	SNOMED
		MTL5302002 Transfer set stay safe	42102117 Transfer set stay safe	Non-standard	EDI			
	6 mon	MTL5302006 Stay safe catheter extension Luer-lock	42100905 Stay safe catheter extension Luer-lock	Non-standard	EDI			
		NA Transfer set	42102116 Transfer set	Non-standard	EDI			
PD related complication								
Peritonitis	2002-2007	K650.000.00 Acute peritonitis	4195847 Acute peritonitis	Standard	SNOMED	Not recommended		
	2004-2010	K659.000.00 Peritonitis, unspecified	196152 Peritonitis	Standard	SNOMED			
	2015-2021	K918.005.00 Peritonitis due to CAPD	200447 Gastrointestinal complication	Standard	SNOMED	44783176 CAPD associated peritonitis	Standard	SNOMED
	2009-2022	T857.004.00 Peritonitis due to CAPD catheter	440276 Infection and/or inflammatory reaction due to internal prosthetic device, implant and/or graft	Standard	SNOMED			
Tunnel infection		T857.004.01 Tunnel infection due to CAPD catheter				4128368 PD catheter tunnel infection	Standard	SNOMED

Exit-site infection	T857.004.02 Exit site infection due to CAPD catheter				4126450 PD catheter exit site infection	Standard	SNOMED
	T856.003 CAPD malposition	43021974 Complication associated with device	Standard	SNOMED	4207135 Malposition of PD catheter	Standard	SNOMED
Outflow failure	T856.001 Displacement of intra-PD catheter				4126451 Migration of PD catheter	Standard	SNOMED
	T856.004 CAPD obstruction, mechanical				4032292 Obstruction of PD catheter	Standard	SNOMED
Pericatheter leak	T856.002 Leakage of intra-PD catheter				4229542 PD leakage	Standard	SNOMED
	K4090.000.04 Inguinal hernia	4288544 Inguinal hernia	Standard	SNOMED	Not recommended		
	K412.000.01 Bilateral femoral hernia	35625035 Bilateral femoral hernia	Standard	SNOMED			
	K419.000.01 Unilateral femoral hernia	4178874 Femoral hernia	Standard	SNOMED			
	K429.000.01 Umbilical hernia	4245842 Umbilical hernia	Standard	SNOMED			
	K432.000.01 Incisional hernia	198464 Incisional hernia	Standard	SNOMED			
Abdominal wall herniation	K439.000.01 Ventral hernia	4188155 Hernia of anterior abdominal wall	Standard	SNOMED			
	K439.001.02 Incisional hernia						
	K439.002.02 Epigastric hernia						
	K439.003.00 Spigelian hernia						
	K469.000.01 Abdominal hernia	4028373 Hernia of abdominal wall	Standard	SNOMED			
	NA	NA			37017448 Abdominal hernia as complication of PD	Standard	SNOMED
Catheter cuff extrusion	NA	NA			4126130 Extrusion of PD catheter cuff	Standard	SNOMED

PD = peritoneal dialysis, CAPD = continuous ambulatory peritoneal dialysis, CDM = common data model, FEEDER-NET = Federated E-Health for Evidence Renovation Network, NA = not-applicable, SNOMED = Systematized Nomenclature of Medicine, EDI = electronic data interchange.

Supplementary Table S2. Comparison of term/codes between SNOMED, study institute, and mapped OMOP-CDM.

Name	SNOMED Concept-ID	Concept code	EHR code	OMOP Concept_ID
Complication of PD	444024	33461007	T80.9	444024
Abdominal hernia as complication of PD	37017448	713724000		
Bloodstained peritoneal dialysis effluent	4128367	236556004		
Encapsulating peritoneal sclerosis associated with PD	4234922	440663004		
Sclerosing peritonitis as complication of PD	37017444	713720009	T857.003	440276
PD associated encapsulating peritoneal sclerosis	44792779	365001000000107		
Hydrothorax as complication of PD	37017445	713721008	J94.8	
Hemoperitoneum as complication of PD	37017451	713727007	K66.1	
Pain during inflow of dialysate	4032289	236554001	R10.2	
Pain during outflow of dialysate	4128366	236555000	R10.2	
Inadequate peritoneal dialysis	45765649	702634004		
Loss of solute clearance	4126128	236560001		
Loss of ultrafiltration	4032290	236559006		
PD-associated peritonitis	4081057	276883000	T857.004	440276
PD catheter exit site infection	4126450	236557008	T857.004.02	440276
PD catheter tunnel infection	4128368	236558003	T857.004.01	440276
Peritoneal dialysis leakage	4229542	404813001		
Peritoneal dialysis access failure	762939	433991000124108		
Mechanical complication of PD	440302	431028002	T856.000.00	43021974
Extrusion of PD catheter cuff	4126130	236566007	T856	
Leakage from Tenckhoff catheter	43021363	473112005		
Leakage of PD catheter	43021418	473190001	T856.002.00	43021974
Malfunction of PD catheter	43021389	473147002		
Malposition of PD catheter	4207135	440551001	T856.003.00	43021974
Migration of Tenckhoff catheter	43021415	473186003		
Migration of PD catheter	4126451	236563004		
Misplacement of acute PD catheter	4126452	236567003		

Obstruction of PD catheter	4032292	236562009	T856.004.0<br>0	43021974

Abbreviations: EHR, electrical health record; ID, identification; SNOMED, systematized nomenclature of medicine; PD, peritoneal dialysis; OMOP, Observational Medical Outcomes Partnership.
